# First‐Line PD‐1 Inhibitor‐Based Therapy for Advanced or Metastatic Esophageal Squamous Cell Carcinoma: A Revised Systematic Review and Meta‐Analysis of Phase III Trials

**DOI:** 10.1002/cnr2.70660

**Published:** 2026-09-06

**Authors:** Vincent Rothweiler, Christoph Roderburg, Muemtaz Koeksal, Björn Erik Ole Jensen, Torsten Feldt, Ilyas Gencer, Johannes C. Fischer, Wilfried Budach, Wolfram Trudo Knoefel, Guangyu Lu, Edwin Bölke

**Affiliations:** ^1^ Medical School of Heinrich Heine University Düsseldorf Düsseldorf Germany; ^2^ Department of Gastroenterology, Hepatology and Infectious Diseases, Medical Faculty and University Hospital Düsseldorf Heinrich Heine University Düsseldorf Düsseldorf Germany; ^3^ Department of Radiation Oncology University Hospital OWL, Klinikum Bielefeld Bielefeld Germany; ^4^ Institute for Transplantation Diagnostics and Cellular Therapeutics Heinrich Heine University Düsseldorf Düsseldorf Germany; ^5^ Department of Surgery Heinrich Heine University Düsseldorf Düsseldorf Germany; ^6^ School of Basic Medical Sciences & School of Public Health, Faculty of Medicine Yangzhou University Yangzhou China

**Keywords:** ESCC, esophageal squamous cell carcinoma, immunotherapy, meta‐analysis, overall survival, PD‐1 inhibitor, PD‐L1

## Abstract

**Background and Purpose:**

Immune checkpoint blockade has changed the first‐line treatment landscape for advanced esophageal squamous cell carcinoma (ESCC), but the magnitude and consistency of benefit across phase III trials require careful synthesis.

**Methods:**

We conducted a PRISMA 2020‐guided systematic review of PubMed, Embase, CENTRAL, and Web of Science (January 1, 2015 to April 30, 2025) for phase III randomized trials comparing first‐line PD‐1 inhibitor‐based therapy with chemotherapy in advanced/metastatic ESCC. The primary endpoint was overall survival (OS). Secondary endpoints were safety and prespecified subgroup outcomes. RoB 2 was used for risk‐of‐bias assessment; certainty of evidence was judged with GRADE. A fixed‐effect inverse‐variance model was prespecified for the primary common‐effect analysis, with random‐effects and sensitivity analyses performed secondarily.

**Results:**

Six eligible phase III trials were included in the primary analysis (KEYNOTE‐590 ESCC subgroup, CheckMate‐648 nivolumab‐plus‐chemotherapy arm, ESCORT‐1st, JUPITER‐06, ORIENT‐15, and RATIONALE‐306). Across the six parent‐trial reports, 4137 patients were randomized overall; for pooling, KEYNOTE‐590 was restricted to the prespecified ESCC subgroup and CheckMate‐648 to the nivolumab‐plus‐chemotherapy comparison. PD‐1 inhibitor‐based therapy significantly improved OS versus chemotherapy alone (pooled HR 0.68, 95% CI: 0.63–0.74; *I* ^2^ = 0%, interpreted as low statistical heterogeneity with caution because only six studies were pooled). Qualitative assessment indicated that the treatment effect was generally larger in PD‐L1‐high populations; however, biomarker‐defined subgroup data were not sufficiently harmonized for formal pooling, and the magnitude of benefit in very low or PD‐L1‐negative disease remains uncertain. Grade ≥ 3 treatment‐related adverse events were common in both arms, whereas immune‐related toxicities were more frequent with PD‐1 inhibitor‐based therapy and required active monitoring.

**Conclusion:**

First‐line PD‐1 inhibitor‐based therapy confers a robust OS benefit in advanced/metastatic ESCC and supports chemoimmunotherapy as a contemporary standard of care. Remaining uncertainties relate mainly to biomarker‐negative disease, assay heterogeneity, and cross‐trial differences in chemotherapy backbone and geographic composition.

## 1 Introduction

Esophageal cancer remains a major global health problem. According to GLOBOCAN 2022, more than half a million new cases and more than 440 000 deaths occur annually worldwide, with a marked geographic concentration in Asia [1]. Esophageal squamous cell carcinoma (ESCC) is the dominant histologic subtype globally and is especially prevalent in East Asia, where tobacco exposure, alcohol consumption, nutritional factors, and other environmental risks contribute substantially to disease burden [1, 2].

For patients with unresectable, recurrent, or metastatic ESCC, prognosis remains poor. Cytotoxic doublets such as fluoropyrimidine/platinum or paclitaxel/platinum have long been accepted first‐line standards, yet median overall survival (OS) with chemotherapy alone is limited, and durable disease control is uncommon [2, 3]. These limitations created a strong rationale for incorporating immune checkpoint inhibition into earlier lines of therapy.

Biologically, ESCC is an attractive candidate for immunotherapy. Compared with esophageal adenocarcinoma, ESCC more often shows an inflamed tumor microenvironment and clinically relevant PD‐L1 expression, although the predictive performance of PD‐L1 remains imperfect and assay‐dependent [2, 4, 5]. During the last few years, several large phase III randomized trials have tested PD‐1 inhibitor‐based strategies in the first‐line setting and have consistently suggested improved survival with chemoimmunotherapy [6, 7, 8, 9, 10, 11, 12].

However, cross‐trial interpretation is not straightforward. Relevant sources of clinical heterogeneity include differences in immunotherapeutic agent, chemotherapy backbone, geography, histologic eligibility, and PD‐L1 assay/cut‐off. In addition, at least one pivotal study enrolled mixed histologies and another enrolled only PD‐L1‐positive disease, which complicates naive pooling. A rigorously updated meta‐analysis focused specifically on advanced ESCC is therefore clinically relevant. The objective of the present study was to synthesize phase III evidence on first‐line PD‐1 inhibitor‐based treatment in advanced or metastatic ESCC, quantify the pooled OS effect, and examine how methodological and clinical heterogeneity influence interpretation.

## 2 Methods

This review was conducted according to the PRISMA 2020 statement [13]. The completed official PRISMA 2020 checklist, with the location of each item reported by manuscript page or Supporting Information Section, is provided in Table S1. The review was not prospectively registered in PROSPERO; the absence of registration has been acknowledged as a limitation. No separate review protocol was prepared or made publicly available. A full reproducible electronic search strategy is provided in Supporting Information Methods S1.

PubMed, Embase, the Cochrane Central Register of Controlled Trials (CENTRAL), and Web of Science were searched from January 1, 2015 through April 30, 2025. Search terms combined controlled vocabulary and free‐text terms for esophageal squamous cell carcinoma, PD‐1 inhibitors, immunotherapy, and randomized phase III trials. Reference lists of relevant reviews and guidelines were checked for completeness.

Eligible studies were phase III randomized controlled trials enrolling adults with untreated unresectable, recurrent, locally advanced, or metastatic ESCC and comparing a PD‐1 inhibitor‐based first‐line regimen against chemotherapy alone. Trials with mixed histologies were eligible only when ESCC‐specific outcomes were reported separately. For multi‐arm studies, only the chemotherapy‐containing PD‐1 experimental arm was extracted for the primary pooled analysis to reduce clinical heterogeneity.

Two reviewers independently screened records, assessed full texts, and extracted data. Discrepancies were resolved by consensus and, when needed, by discussion with a senior author. Extracted variables included trial design, eligibility criteria, chemotherapy backbone, biomarker definition, sample size, and published hazard ratios (HRs) with confidence intervals (CIs) for OS. Secondary outcomes were grade ≥ 3 treatment‐related adverse events, reported immune‐related adverse events, and prespecified subgroup outcomes.

Risk of bias was assessed independently by two reviewers with the Cochrane RoB 2 tool [14]. Judgments were made for bias arising from the randomization process, deviations from intended interventions, missing outcome data, measurement of the outcome, and selection of the reported result. Certainty of evidence was evaluated with the GRADE framework [15].

The primary endpoint was OS. Log‐transformed HRs and standard errors were derived from published confidence intervals. A fixed‐effect inverse‐variance model was prespecified for the primary common‐effect analysis because the primary dataset was intentionally restricted to first‐line phase III PD‐1 inhibitor plus chemotherapy comparisons against chemotherapy alone. This choice was not based on an assumption that all studies were clinically identical; therefore, random‐effects analyses and sensitivity analyses were also performed to test robustness against clinical and methodological diversity.

Heterogeneity was summarized with the *I* ^2^ statistic and interpreted according to the rough guidance in the Cochrane Handbook: 0%–40% might not be important, 30%–60% may represent moderate heterogeneity, 50%–90% may represent substantial heterogeneity, and 75%–100% may represent considerable heterogeneity [16]. Because *I* ^2^ is imprecise when fewer than 10 studies are pooled, heterogeneity judgments were interpreted cautiously and in relation to the direction and overlap of individual study estimates. Formal assessment of small‐study effects or publication bias (e.g., funnel‐plot asymmetry or Egger testing) was not performed because fewer than 10 studies were available, making such analyses unreliable.

PD‐L1‐defined subgroup outcomes and safety endpoints were planned as secondary outcomes. Because PD‐L1 assays and cut‐offs differed materially across trials, including combined positive score (CPS), tumor proportion score (TPS)/tumor‐cell PD‐L1 expression, tumor‐area positivity (TAP), and trial‐specific exploratory thresholds, biomarker‐defined subgroups were synthesized qualitatively rather than pooled quantitatively. Similarly, adverse‐event outcomes were synthesized narratively because event definitions, immune‐related toxicity categories, and reporting granularity were not fully harmonized across trials. The revised statistical analysis was conducted in Python 3.13 using SciPy 1.17.0 for inverse‐variance calculations and Matplotlib 3.10.8 for figure generation.

## 3 Results

The database searches identified 416 records (PubMed *n* = 96, Embase *n* = 168, CENTRAL *n* = 34, Web of Science *n* = 118). After removal of 151 duplicates, 265 records were screened, 244 were excluded at title/abstract stage, and 21 reports were assessed for eligibility. Fourteen reports were excluded after full‐text review. The final revised review set comprised seven studies: six studies in the primary pooled meta‐analysis and one additional biomarker‐restricted study used only in sensitivity analysis (Table 1). The study selection process is summarized in (Figure S1 and Table S2).

**TABLE 1:** Study design and baseline characteristics of included phase III trials.

Trial	Design/population	Experimental versus control regimen	PD‐L1 metric/selection	Randomized n	Key baseline characteristics	Extracted OS HR
KEYNOTE‐590 [6]	Double‐blind, global; untreated advanced/metastatic esophageal cancer; prespecified ESCC subgroup extracted	Pembrolizumab + cisplatin/5‐FU versus placebo + cisplatin/5‐FU	CPS; key cut‐off ≥ 10	373/376; 548 ESCC	Age 64/62 years; male 82.0%/84.8%; ECOG 0 39.9%/39.9%^b^	0.72 (0.60–0.88)
CheckMate‐648 [7]	Open‐label, global; untreated unresectable/recurrent/metastatic ESCC	Nivolumab + fluoropyrimidine/cisplatin versus chemotherapy	Tumor‐cell PD‐L1; cut‐off ≥ 1%	321/324	Age 64/64 years; male 79%/85%; ECOG 0 46%/47%	0.74 (0.58–0.96)
ESCORT‐1st [8]	Double‐blind; China; untreated advanced/metastatic ESCC	Camrelizumab + paclitaxel/cisplatin versus placebo + paclitaxel/cisplatin	Exploratory analyses	298/298	Age 62/62 years; male 87.2%/88.3%; ECOG 0 23.8%/22.1%	0.70 (0.56–0.88)
JUPITER‐06 [9]	Double‐blind; China; untreated advanced ESCC	Toripalimab + paclitaxel/cisplatin versus placebo + paclitaxel/cisplatin	Exploratory analyses	257/257	Age 63/62 years; male 84.4%/85.6%; ECOG 0 25.7%/26.5%	0.58 (0.42–0.78)
ORIENT‐15 [10]	Double‐blind; predominantly China; untreated locally advanced/metastatic ESCC	Sintilimab + cisplatin/paclitaxel or cisplatin/5‐FU versus placebo + chemotherapy	CPS; key cut‐off ≥ 10	327/332	Age 63/63 years; male 85%/87%; ECOG 0 24%/24%	0.63 (0.51–0.78)
RATIONALE‐306 [11]	Double‐blind, global; untreated advanced/metastatic ESCC	Tislelizumab + investigator‐choice platinum doublet versus placebo + chemotherapy	Exploratory; later TAP analyses	326/323	Age 64/65 years; male 86.5%/87.0%; ECOG 0 33.4%/32.2%	0.66 (0.54–0.80)
ASTRUM‐007^a^ [12]	Double‐blind; China; untreated PD‐L1‐positive (CPS ≥ 1) advanced/metastatic ESCC	Serplulimab + cisplatin/5‐FU versus placebo + cisplatin/5‐FU	Eligibility restricted to CPS ≥ 1	368/183	Age 64/64 years; male 86.1%/83.6%; ECOG 0 25.3%/29.0%	0.68 (0.53–0.87)

Abbreviations: CPS, combined positive score; ECOG, Eastern Cooperative Oncology Group; ESCC, esophageal squamous cell carcinoma; HR, hazard ratio; OS, overall survival; TAP, tumor‐area positivity; TPS, tumor proportion score.

^a^ ASTRUM‐007 was included only in sensitivity analysis because enrollment was restricted to PD‐L1 CPS ≥ 1. GEMSTONE‐304 was not included because it investigated PD‐L1 blockade rather than PD‐1 blockade. Baseline values are shown as experimental/control.

^b^ KEYNOTE‐590 baseline characteristics refer to the parent intention‐to‐treat population because ESCC‐subgroup baseline characteristics were not reported separately.

The primary analysis included six phase III trials: KEYNOTE‐590 (ESCC subgroup), CheckMate‐648 (nivolumab plus chemotherapy arm), ESCORT‐1st, JUPITER‐06, ORIENT‐15, and RATIONALE‐306 [6, 7, 8, 9, 10, 11]. Across the six parent‐trial reports, 4137 patients were randomized overall; for pooling, only the relevant ESCC/comparator data were extracted where applicable. For KEYNOTE‐590, only the prespecified ESCC subgroup estimate was extracted because the overall trial population included adenocarcinoma [6]. One additional trial, ASTRUM‐007, met most clinical criteria but enrolled only PD‐L1‐positive disease (CPS ≥ 1); it was therefore incorporated as a sensitivity analysis rather than the primary pooled dataset [12]. GEMSTONE‐304 was not included because it investigated PD‐L1 blockade rather than PD‐1 blockade.

**FIGURE 1:**
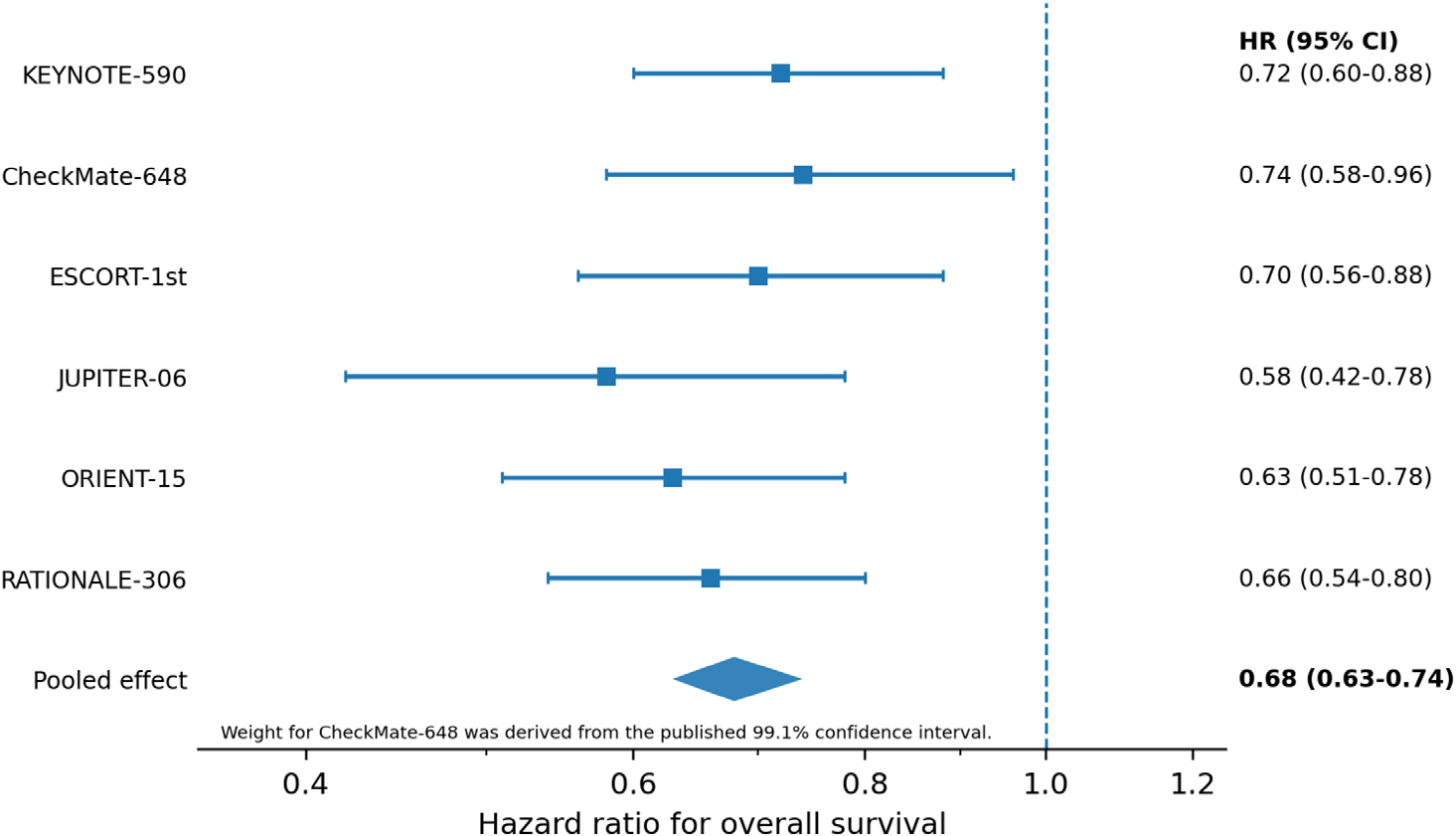
Forest plot of the primary pooled OS analysis. Primary fixed‐effect inverse‐variance meta‐analysis of six phase III comparisons evaluating first‐line PD‐1 inhibitor‐based therapy versus chemotherapy in advanced/metastatic ESCC. Pooled HR 0.68 (95% CI: 0.63–0.74); *I* ^2^ = 0%, interpreted as low statistical heterogeneity with caution because of the limited number of trials.

All six primary‐analysis trials reported an OS advantage in favor of PD‐1 inhibitor‐based therapy. Individual published HRs were 0.72 for the KEYNOTE‐590 ESCC subgroup, 0.74 for nivolumab plus chemotherapy in CheckMate‐648, 0.70 for ESCORT‐1st, 0.58 for JUPITER‐06, 0.63 for ORIENT‐15, and 0.66 for RATIONALE‐306 [6, 7, 8, 9, 10, 11]. The pooled fixed‐effect estimate demonstrated a significant OS benefit for PD‐1 inhibitor‐based therapy versus chemotherapy alone (HR 0.68, 95% CI: 0.63–0.74), corresponding to an approximately 32% relative reduction in the hazard of death. Statistical heterogeneity was low by *I* ^2^ (*I* ^2^ = 0%) (Figure 1).

A sensitivity analysis incorporating ASTRUM‐007 yielded an almost identical result (HR 0.68, 95% CI: 0.63–0.74; *I* ^2^ = 0%), indicating that the main finding was not materially altered by inclusion of the biomarker‐enriched study [12]. A secondary random‐effects analysis produced the same rounded pooled estimate because the estimated between‐study variance was zero; nevertheless, this result was interpreted cautiously because *I* ^2^ has limited precision when fewer than 10 studies are available. Consistent with the prespecified methodological approach, reporting bias was not formally assessed because only six studies contributed to the primary meta‐analysis.

Biomarker‐defined subgroup findings were synthesized qualitatively. Subgroup reporting was generally directionally consistent with the overall findings, but harmonized quantitative pooling was not feasible. First, PD‐L1 was assessed differently across trials. Second, the clinically important PD‐L1‐negative subgroup was incompletely and inconsistently reported. For example, KEYNOTE‐590 suggested attenuation of benefit in patients with PD‐L1 CPS < 10, whereas ORIENT‐15 reported no clear benefit in the small CPS < 1 subgroup; CheckMate‐648 reported results by TPS/tumor‐cell PD‐L1 expression rather than CPS, and RATIONALE‐306 incorporated TAP‐based PD‐L1 analyses in its reporting framework [6, 7, 10, 11]. Accordingly, subgroup results were interpreted qualitatively.

Safety findings were clinically important but methodologically heterogeneous. In the pivotal trials, grade ≥ 3 treatment‐related adverse events were common in both experimental and control groups because chemotherapy was part of all regimens in the primary analysis. Reported grade ≥ 3 treatment‐related adverse events ranged from 47% to 72% in PD‐1 inhibitor‐based arms and from 36% to 68% in control arms [6, 7, 8, 9, 10, 11]. Immune‐related adverse events such as hypothyroidism, rash, hepatitis, pneumonitis, and colitis were more frequent with PD‐1 inhibitor therapy, but most were manageable with established treatment algorithms. These findings support the view that improved survival is achieved at the expense of greater immune‐mediated toxicity monitoring rather than a dramatic increase in overall severe toxicity.

RoB 2 assessment showed low overall risk of bias for five of the six primary‐analysis studies and some concerns for CheckMate‐648, mainly related to its open‐label design and potential deviations from intended interventions. ASTRUM‐007 was judged at low risk of bias for the sensitivity analysis. Detailed domain‐level judgments are provided in Table S3.

GRADE certainty was high for the primary OS outcome because the evidence consisted of phase III randomized data with consistent direction of effect, a precise pooled estimate, and low statistical heterogeneity by *I* ^2^. Certainty for safety was moderate because adverse‐event definitions and reporting were not fully harmonized. Certainty for PD‐L1‐low/negative subgroup interpretation was low to moderate owing to indirectness and imprecision from varying assays, cut‐offs, and small subgroup sizes. Detailed GRADE judgments are provided in Table S4.

## 4 Discussion

This revised meta‐analysis demonstrates a robust survival advantage for first‐line PD‐1 inhibitor‐based therapy in advanced or metastatic ESCC, with a pooled OS HR of 0.68 and low statistical heterogeneity by *I* ^2^. The magnitude of effect is clinically meaningful and aligns with the direction and size of benefit observed in each individual phase III trial [6, 7, 8, 9, 10, 11]. Our results also remain stable when the biomarker‐restricted ASTRUM‐007 study is included in sensitivity analysis [12].

These findings are broadly consistent with prior secondary analyses and meta‐analyses, but the present revision strengthens the evidence base by correcting extraction issues, clarifying histology‐specific handling, and explicitly addressing clinical heterogeneity [4, 5]. In particular, the current analysis avoids using mixed‐histology overall estimates from KEYNOTE‐590 and avoids conflating the nivolumab‐plus‐ipilimumab arm of CheckMate‐648 with chemotherapy‐containing first‐line regimens, both of which can distort interpretation [6, 7].

The discussion of biomarkers requires caution. Although benefit was generally larger in PD‐L1‐high populations, PD‐L1 did not function as a universal binary gatekeeper. At the same time, the evidence base is weakest in biomarker‐negative disease because subgroup definitions vary and truly PD‐L1‐negative populations are often small or inconsistently reported [6, 7, 8, 9, 10, 11]. This nuance is clinically important: the data clearly support first‐line chemoimmunotherapy in advanced ESCC overall, but they do not fully resolve which patients with very low or negative PD‐L1 expression derive sufficient benefit to justify treatment in every regulatory context.

This interpretation is strengthened by the recent meta‐analysis by Beshr et al., which incorporated CPS subgroup data presented by trial investigators at the September 2024 FDA Oncologic Drugs Advisory Committee meeting and evaluated CPS thresholds including < 10, < 5, and < 1 [17]. In that analysis, first‐line PD‐1/PD‐L1 inhibitor‐based therapy produced an overall OS benefit consistent with our pooled estimate, but OS benefit was not evident in the CPS < 1 subgroup and was greatest in CPS ≥ 10 disease [17]. These external data support a cautious, assay‐aware interpretation: the aggregate first‐line benefit is robust, but the net benefit in strictly PD‐L1‐negative ESCC remains uncertain and may be less favorable.

Clinical heterogeneity across trials should be acknowledged. The included studies used different PD‐1 antibodies, chemotherapy backbones, geographic recruitment patterns, and PD‐L1 scoring systems. Most studies tested chemoimmunotherapy, whereas CheckMate‐648 also included a chemotherapy‐free dual‐checkpoint arm not pooled here. These differences matter because they may influence absolute survival, tolerability, and regulatory interpretation even when the relative treatment effect appears consistent [6, 7, 8, 9, 10, 11, 12].

The safety profile deserves explicit clinical framing. Severe treatment‐related adverse events remain common in advanced ESCC regardless of treatment assignment because platinum‐based doublets are inherently toxic. The distinctive contribution of PD‐1 inhibitors is the addition of immune‐mediated adverse events rather than a wholesale change in the overall toxicity burden. For practicing clinicians, this means that survival gains are accompanied by a need for earlier recognition of endocrine, pulmonary, hepatic, and gastrointestinal immune‐related toxicities, multidisciplinary collaboration, and patient education.

The present analysis has direct clinical implications. First, it supports PD‐1 inhibitor‐based chemoimmunotherapy as a frontline standard for fit patients with advanced ESCC. Second, it suggests that biomarker interpretation should remain contextual and assay‐specific rather than reductionist. Third, it underlines the importance of discussing treatment choice in relation to chemotherapy backbone, regulatory label, and local access. Contemporary guidelines already reflect this shift toward frontline immunotherapy‐based treatment, but they differ in how strongly they emphasize PD‐L1 thresholds for specific agents [3].

Strengths of this revision include restriction to phase III randomized evidence, explicit histology‐focused data extraction, formal RoB 2 assessment, transparent GRADE judgments, and sensitivity analyses that test the impact of biomarker‐enriched studies. The main limitations are equally important: this is a literature‐based rather than individual‐patient‐data meta‐analysis; subgroup reporting was insufficiently harmonized for formal pooled biomarker analyses; safety endpoints were heterogeneous and therefore synthesized narratively; *I* ^2^ is imprecise in small meta‐analyses; and the review was not prospectively registered in PROSPERO. In addition, at least two pivotal trial reports had published journal corrections, which were checked and incorporated into the updated reference list; no retractions were identified among the key primary trial publications.

## 5 Conclusion

In advanced or metastatic ESCC, first‐line PD‐1 inhibitor‐based therapy provides a consistent and clinically meaningful OS advantage over chemotherapy alone. The benefit is robust across modern phase III trials and supports frontline chemoimmunotherapy as a standard therapeutic approach. Remaining gaps concern biomarker‐negative disease, cross‐trial assay heterogeneity, and optimal tailoring of regimen selection in routine practice.

## Author Contributions

**Vincent Rothweiler:** conceptualization, methodology, investigation, data curation, formal analysis, visualization, writing – original draft, writing – review and editing. **Guangyu Lu:** writing – original draft. **Muemtaz Koeksal:** writing – original draft. **Björn Erik Ole Jensen:** writing – original draft. **Johannes C. Fischer:** writing – original draft, methodology. **Edwin Bölke:** conceptualization, writing – original draft, investigation, writing – review and editing. **Wilfried Budach:** methodology, writing – original draft. **Ilyas Gencer:** investigation, writing – original draft. **Christoph Roderburg:** writing – original draft. **Wolfram Trudo Knoefel:** writing – original draft. **Torsten Feldt:** writing – original draft.

## Funding

The authors have nothing to report.

## Conflicts of Interest

The authors declare no conflicts of interest.

## Supporting information


**Table S1:** PRISMA 2020 checklist.
**Figure S1:** PRISMA 2020 flow diagram.
**Table S2:** Study selection counts.
**Table S3:** RoB 2 summary.
**Table S4:** GRADE summary.

## Data Availability

No individual patient data were used. All data were extracted from publicly available published trial reports. The extracted study‐level dataset and analytic code are available from the corresponding author upon reasonable request.
