# Supplementary material for: First‐Line PD‐1 Inhibitor‐Based Therapy for Advanced or Metastatic Esophageal Squamous Cell Carcinoma: A Revised Systematic Review and Meta‐Analysis of Phase III Trials
Source: Cancer Rep (Hoboken). 2026 Sep 6;9(9):e70660. doi: 10.1002/cnr2.70660 (PMC13547580; doi:10.1002/cnr2.70660)
Supplement: Supplementary file 1 [file CNR2-9-e70660-s001.docx]

**Supplementary Appendix
Revised ESCC systematic review and meta-analysis**

Revised draft prepared for peer-review response and second revision.

# Supplementary Methods S1. Reproducible electronic search strategy

Databases searched: PubMed, Embase, CENTRAL, and Web of Science. Search date: April 30, 2025. Time window: January 1, 2015 to April 30, 2025. Language restriction: English.

PubMed: ("Esophageal Squamous Cell Carcinoma"[Title/Abstract] OR "Oesophageal Squamous Cell Carcinoma"[Title/Abstract] OR ESCC[Title/Abstract] OR ("Esophageal Neoplasms"[Mesh] AND squamous[Title/Abstract])) AND (pembrolizumab OR nivolumab OR camrelizumab OR tislelizumab OR sintilimab OR toripalimab OR serplulimab OR "PD-1" OR "programmed cell death 1") AND (randomized OR randomised OR "phase III" OR "phase 3") AND ("first-line" OR untreated OR treatment-naive OR treatment-naïve OR advanced OR metastatic). Filters: English; 2015/01/01 to 2025/04/30.

Embase: Equivalent Emtree/free-text strategy combining ESCC terms, PD-1 inhibitor terms, and phase III randomized first-line trial terms; limited to English-language records from 2015 to April 30, 2025.

CENTRAL: ESCC AND (PD-1 OR pembrolizumab OR nivolumab OR camrelizumab OR tislelizumab OR sintilimab OR toripalimab OR serplulimab) AND randomized AND first-line.

Web of Science: TS=(esophageal squamous cell carcinoma OR oesophageal squamous cell carcinoma OR ESCC) AND TS=(pembrolizumab OR nivolumab OR camrelizumab OR tislelizumab OR sintilimab OR toripalimab OR serplulimab OR PD-1) AND TS=(phase III OR phase 3 OR random*) AND TS=(first-line OR untreated OR advanced OR metastatic).

Supplementary note on protocol registration: This review was not prospectively registered in PROSPERO. The absence of prospective registration has been stated in the main manuscript and treated as a limitation.

# Supplementary Table S1. PRISMA 2020 checklist

| **PRISMA item** | **Topic** | **Location in manuscript** | **Notes** |
| --- | --- | --- | --- |
| 1 | Title | Title page | Identifies the report as a systematic review and meta-analysis. |
| 2 | Abstract | Abstract | Structured abstract includes background, methods, results, and conclusion. |
| 3 | Rationale | Introduction | Rationale for updated ESCC-specific phase III synthesis. |
| 4 | Objectives | Introduction | Objective stated at end of Introduction. |
| 5 | Eligibility criteria | Methods | Eligibility criteria for population, intervention, comparator, design, and histology. |
| 6 | Information sources | Methods; Supplementary Methods S1 | Databases and date range reported. |
| 7 | Search strategy | Supplementary Methods S1 | Full reproducible strategy provided. |
| 8 | Selection process | Methods; Results; Supplementary Figure S1/Table S2 | Two reviewers screened records and assessed full texts. |
| 9 | Data collection process | Methods | Two reviewers extracted data independently. |
| 10a | Data items - outcomes | Methods | Primary OS, secondary safety and subgroup outcomes specified. |
| 10b | Data items - other variables | Methods/Table 1 | Trial design, regimen, biomarker definition, sample size, HRs extracted. |
| 11 | Risk of bias assessment | Methods; Results; Supplementary Table S3 | RoB 2 tool and results reported. |
| 12 | Effect measures | Methods | Hazard ratios for OS. |
| 13a | Synthesis eligibility | Methods/Results | Studies eligible for each synthesis specified. |
| 13b | Data preparation | Methods | Log HRs and SEs derived from published CIs. |
| 13c | Tabulation/visual display | Table 1; Figure 1; Supplementary Figure S1 | Study characteristics, forest plot, and PRISMA flow shown. |
| 13d | Synthesis methods | Methods | Fixed-effect primary common-effect analysis; random-effects and sensitivity analyses. |
| 13e | Exploration of heterogeneity | Methods/Results | I2 guidance and sensitivity analysis reported. |
| 13f | Sensitivity analyses | Methods/Results | ASTRUM-007 and random-effects analyses reported. |
| 14 | Reporting bias assessment | Methods/Discussion | Not formally assessed due small number of trials; discussed as limitation. |
| 15 | Certainty assessment | Methods; Results; Supplementary Table S4 | GRADE used and summarized. |
| 16a | Study selection results | Results; Supplementary Figure S1/Table S2 | Records, exclusions, and included studies reported. |
| 16b | Excluded studies | Results; Supplementary Table S2 | GEMSTONE-304 reason stated. |
| 17 | Study characteristics | Table 1 | Characteristics of included studies reported. |
| 18 | Risk of bias in studies | Results; Supplementary Table S3 | RoB 2 results reported. |
| 19 | Results of individual studies | Results; Figure 1 | Individual HRs and CIs reported. |
| 20a | Results of syntheses | Results; Figure 1 | Pooled HR and heterogeneity reported. |
| 20b | Statistical heterogeneity | Results | I2 reported and interpreted cautiously. |
| 20c | Investigations of heterogeneity | Results/Discussion | Sensitivity analysis and qualitative subgroup interpretation. |
| 20d | Sensitivity analyses results | Results | ASTRUM-007 sensitivity analysis reported. |
| 21 | Reporting biases | Discussion | Formal assessment not possible due few studies; limitation acknowledged. |
| 22 | Certainty of evidence | Results; Supplementary Table S4 | GRADE findings summarized. |
| 23a | Discussion - interpretation | Discussion | Interpretation in context of evidence and biomarkers. |
| 23b | Discussion - limitations of evidence | Discussion | Limitations of literature-based analysis, subgroup and safety reporting, I2, and PROSPERO. |
| 23c | Discussion - limitations of review process | Discussion | Non-registration and literature-based limitations acknowledged. |
| 23d | Discussion - implications | Discussion/Conclusion | Clinical implications and remaining uncertainties. |
| 24a | Registration | Methods/Discussion | Not prospectively registered in PROSPERO. |
| 24b | Protocol availability | Methods | No protocol available. |
| 24c | Protocol amendments | Not applicable | No registered protocol. |
| 25 | Support | Funding | No external funding. |
| 26 | Competing interests | Conflict of interest | No conflict declared. |
| 27 | Availability of data/code/materials | Data availability | Public trial reports; no individual patient data used. |

# Supplementary Figure S1. PRISMA 2020 flow diagram

Figure legend. PRISMA flow diagram for the revised search and study selection process.

# Supplementary Table S2. Study selection counts

| **Flow item** | **Count** |
| --- | --- |
| Records identified from PubMed | 96 |
| Records identified from Embase | 168 |
| Records identified from CENTRAL | 34 |
| Records identified from Web of Science | 118 |
| Total database records identified | 416 |
| Duplicate records removed | 151 |
| Records screened | 265 |
| Records excluded at title/abstract stage | 244 |
| Reports sought for retrieval | 21 |
| Reports not retrieved | 0 |
| Reports assessed for eligibility | 21 |
| Reports excluded after full-text review | 14 |
| Studies included in revised review set | 7 |
| Primary-analysis studies included in pooled meta-analysis | 6 |
| Additional biomarker-restricted study used in sensitivity analysis (ASTRUM-007) | 1 |
| Clearly ineligible first-line trial at full-text stage because it evaluated PD-L1 rather than PD-1 blockade (GEMSTONE-304) | 1 |

# Supplementary Table S3. RoB 2 summary

| **Study** | **Randomization** | **Deviations** | **Missing data** | **Outcome measurement** | **Reported result** | **Overall** |
| --- | --- | --- | --- | --- | --- | --- |
| KEYNOTE-590 (ESCC subgroup) | Low | Low | Low | Low | Low | Low |
| CheckMate-648 (nivo+chemo arm) | Low | Some concerns | Low | Low | Low | Some concerns |
| ESCORT-1st | Low | Low | Low | Low | Low | Low |
| JUPITER-06 | Low | Low | Low | Low | Low | Low |
| ORIENT-15 | Low | Low | Low | Low | Low | Low |
| RATIONALE-306 | Low | Low | Low | Low | Low | Low |
| ASTRUM-007 (sensitivity analysis) | Low | Low | Low | Low | Low | Low |

# Supplementary Table S4. GRADE summary

| **Outcome** | **Certainty** | **Rationale** |
| --- | --- | --- |
| Overall survival (primary pooled outcome) | High | Phase III randomized evidence; consistent direction and magnitude of effect; precise pooled estimate; I2=0%, interpreted cautiously as low statistical heterogeneity because only six studies were pooled. |
| Safety (narrative synthesis) | Moderate | Randomized evidence, but downgraded for inconsistency because adverse-event definitions and reporting were not fully harmonized. |
| PD-L1-low/negative subgroups | Low to Moderate | Downgraded for indirectness and imprecision because assays, cut-offs, and subgroup definitions differed materially across trials and some subgroups were small. |
